# Adipose Stem Cell-Based Treatments for Wound Healing

**DOI:** 10.3389/fcell.2021.821652

**Published:** 2022-01-11

**Authors:** Ning Zeng, Hongbo Chen, Yiping Wu, Zeming Liu

**Affiliations:** Department of Plastic and Cosmetic Surgery, Tongji Hospital, Tongji Medical College, Huazhong University of Science and Technology, Wuhan, China

**Keywords:** adipose stem cells, wound healing, regenerative medicine, skin regeneration, inflammation

## Abstract

Wound healing is one of the most complex physiological regulation mechanisms of the human body. Stem cell technology has had a significant impact on regenerative medicine. Adipose stem cells (ASCs) have many advantages, including their ease of harvesting and high yield, rich content of cell components and cytokines, and strong practicability. They have rapidly become a favored tool in regenerative medicine. Here, we summarize the mechanism and clinical therapeutic potential of ASCs in wound repair.

## Introduction

The skin is the largest organ of the body. It is a key structure that protects internal tissues from mechanical damage, microbial infection, ultraviolet radiation, and extreme temperatures (Falanga, 2005; Ren et al., 2019; Rodrigues et al., 2019; Yang et al., 2020). In the United States, the annual medical cost of adverse wounds, including surgical incisions and scars, is $12 billion (Fife and Carter, 2012; Leavitt et al., 2016). Wound healing is a highly complex physiological regulation mechanism (Rodrigues et al., 2019) and a sophisticated multicellular process involving the coordination of various cell types and cytokines (Ho Jeong, 2010). Interactions involving epidermal and dermal cells, extracellular matrix (ECM), cytokines, and growth factors coordinate the entire repair process, which can be roughly divided into three stages: inflammation, new tissue formation, and reconstruction (Heublein et al., 2015; Rodrigues et al., 2019). The inflammatory stage includes neutrophil and monocyte recruitment and macrophage activation (Park and Barbul, 2004; Larouche et al., 2018). New tissue formation mainly refers to the proliferation, migration, and recombination of endothelial cells to form new blood vessels. When new blood vessels are formed, resident fibroblasts proliferate and invade fibrin clots to form contractile granulation tissue and produce collagen (Heng, 2011; Ansell and Izeta, 2015; Morikawa et al., 2019). This is followed by the proliferation of epidermal stem cells to rebuild the epidermis and stem cells from sebaceous glands, sweat glands, and hair follicles to form epidermal attachments.

### Routine Treatment of Wounds

In view of the complex, multi-stage, physiological and pathological processes of acute and chronic skin wound healing, efficient targeted wound healing treatment methods have been studied and applied. Thorough surgical debridement, prevention of infection, and elimination of dead spaces can minimize the risk of poor wound healing. Emerging technologies, such as those based on growth factors, bioactive molecules, and gene modification, can also overcome the limitations of wound healing technology to some extent and serve as personalized therapeutic strategies (Tottoli et al., 2020).

However, despite these efforts, existing interventions for wound healing have not been sufficiently effective. While there are several treatments available for both acute and chronic wounds, traditional approaches have had limited success. Due to the limitations of traditional methods, such as drug-based therapy, more effective treatments are needed. Skin regeneration therapy strategies and experimental techniques for cell and tissue engineering have also emerged. Stem cell-based therapy has opened a new door for wound repair and has attracted extensive attention in the field of regenerative medicine.

### Stem Cells

There are thousands of cells undergoing constant daily dynamic changes, such as loss and self-renewal, to maintain tissue homeostasis. Self-renewal is mainly driven by stem cells. Stimulation from regeneration signals, such as the accumulation of crosstalk with niche factors or environmental changes at the time of injury, can disrupt tissue homeostasis, change stem cell behavior, induce self-renewal, and promote tissue growth (Hsu et al., 2011; Cosgrove et al., 2014; Porpiglia et al., 2017). When homeostasis is restored, differentiated progeny can return to their niche, preventing further proliferation and tissue regeneration, and this process is regulated by a careful balance of time-coordinated cell interactions and molecular feedback loops (Fuchs and Blau, 2020).

Stem cells can be divided into embryonic and adult stem cells according to their developmental stage. Embryonic stem cells refer to cells derived from the embryonic inner cell mass or primordial germ cells *in vitro*. Embryonic stem cells have developmental totipotency and can differentiate into any type of cell. Embryonic stem cells can be extensively amplified, screened, frozen, and resuspended *in vitro* without them losing their original characteristics (VanOudenhove et al., 2017; Wang et al., 2019; Sun et al., 2021). Adult stem cells, which are found in various tissues and organs of the body, are undifferentiated cells in a differentiated tissue that can self-renew and differentiate into the specialized cells composing that tissue. These stem cells include hematopoietic stem cells, bone marrow mesenchymal stem cells, neural stem cells, muscle satellite cells, epidermal stem cells, and adipose stem cells (ASCs) (Cinat et al., 2021; Menche and Farin, 2021). In this review, we focus on ASCs.

### Sources and Applications of ASCs

Adipose tissue is a multifunctional tissue that contains a variety of cell types, such as the stromal vascular fraction and mature adipose cells. Stromal vascular fragments (SVFs) are a rich source of ASCs that can be easily isolated from human fat (Whiteside, 2008; O’Halloran et al., 2017). The Mesenchymal and Tissue Stem Cell Committee of the International Society for Cellular Therapy (ISCT MSC) proposes minimal criteria to define human MSC follows: First, MSC must be plastic-adherent when maintained in standard culture conditions. Second, MSC must express CD105, CD73 and CD90, and lack expression of CD45, CD34, CD14 or CD11b, CD79alpha or CD19 and HLA-DR surface molecules. Third, MSC must differentiate to osteoblasts, adipocytes and chondroblasts *in vitro*. ASCs conform to most of the mesenchymal criteria of ISCT MSC, defined as CD45^−^CD235a^−^CD31^−^CD34^+^. The phenotype of cultured ASCs is CD13^+^CD73^+^CD90^+^CD105^+^CD31^−^CD45^−^CD235a^−^ (Dominici et al., 2006; Bourin et al., 2013).

ASCs have many advantages. They can be directly extracted from the adipose layer of a patient. Adipose tissue has a high frequency of stem cells, and ASCs can be used immediately with primary cells without the need for culture amplification. In addition, ASCs provide not only cellular components, but also a large number of cytokines. Currently, ASCs have various clinical applications, including in scar reshaping and tissue repair, regeneration, and reconstruction, which are treatments often associated with cancer and metabolic diseases (Brayfield et al., 2010; Gir et al., 2012; Rodrigues et al., 2014; Strong et al., 2015; Clevenger et al., 2016; Gentile and Garcovich, 2019; Sabol et al., 2019; Qin et al., 2020). Skin repair/regeneration is one of the most common clinical applications of ASCs, which has a positive therapeutic effect when used to treat skin wounds in patients with diabetes, vascular dysfunction, radiation history, or burn history.

### Mechanism of ASCs in Wound Healing

#### Factors Secreted by ASCs

The mechanisms of wound healing by ASCs are complex and diverse. ASCs are involved throughout the entire process of wound healing, including inflammation, proliferation, and remodeling (Hyldig et al., 2017). During inflammation, ASCs may induce the transformation of the macrophage phenotype from pro-inflammatory M1 to anti-inflammatory M2 to regulate inflammation (Lo Sicco et al., 2017). During proliferation and remodeling, ASCs secrete biological factors such as VEGF, HGF, IGF, PDGF, and TGF-β, which promote the proliferation and migration of fibroblasts, the growth of new blood vessels, and the synthesis of collagen and other ECM proteins, which have beneficial effects on the skin (Rehman et al., 2004; Ho Jeong, 2010; Rodrigues et al., 2014; Na et al., 2017). For example, radiation damage to the skin can cause progressive occlusive endarteritis in local tissues, leading to severe tissue ischemia. Mesenchymal stem cells can be used to repair cellular damage and regenerate new blood vessels in ischemic tissues in patients with radioactive skin injury (Bensidhoum et al., 2005; François et al., 2006). ASC replacement after radiotherapy may reduce the incidence of radiation-related skin complications and is used for the prevention and treatment of skin injury related to tumor radiotherapy (Rigotti et al., 2007).

In addition, ASCs inhibit ECM degradation by increasing the binding of matrix metalloproteinases and secreting tissue metalloproteinase inhibitors (Lozito et al., 2014). Proteins in the ECM, in turn, protect against degradation of growth factors and cytokines produced by activated platelets and macrophages, such as PDGF and TGF-β (Barrientos et al., 2008). Finally, *in vitro* studies have confirmed that ASCs may promote re-epithelialization by regulating keratinocyte proliferation and migration (Riis et al., 2017). In summary, ASCs can promote wound healing by reducing inflammation, inducing angiogenesis, promoting the growth of fibroblasts and keratinocytes, and generating ECM.

#### ASC-Derived Extracellular Vesicles

Recent studies have shown that paracrine factors significantly promote the effect of stem cells during tissue repair and that extracellular vesicles may play an important role. Extracellular vesicles include exosomes and microvesicles, which play an important role in and are considered mediators of intercellular communication (Shao et al., 2018; Théry et al., 2018). The differences between exosomes and microvesicles in terms of physical function are yet to be clarified. Microvesicles are large vesicles (50–1000 nm in diameter) that germinate outward from the plasma membrane, whereas exosomes are small vesicles (50–150 nm in diameter), and their secretion requires the fusion of multiple vesicles with the plasma membrane.

**FIGURE 1:**
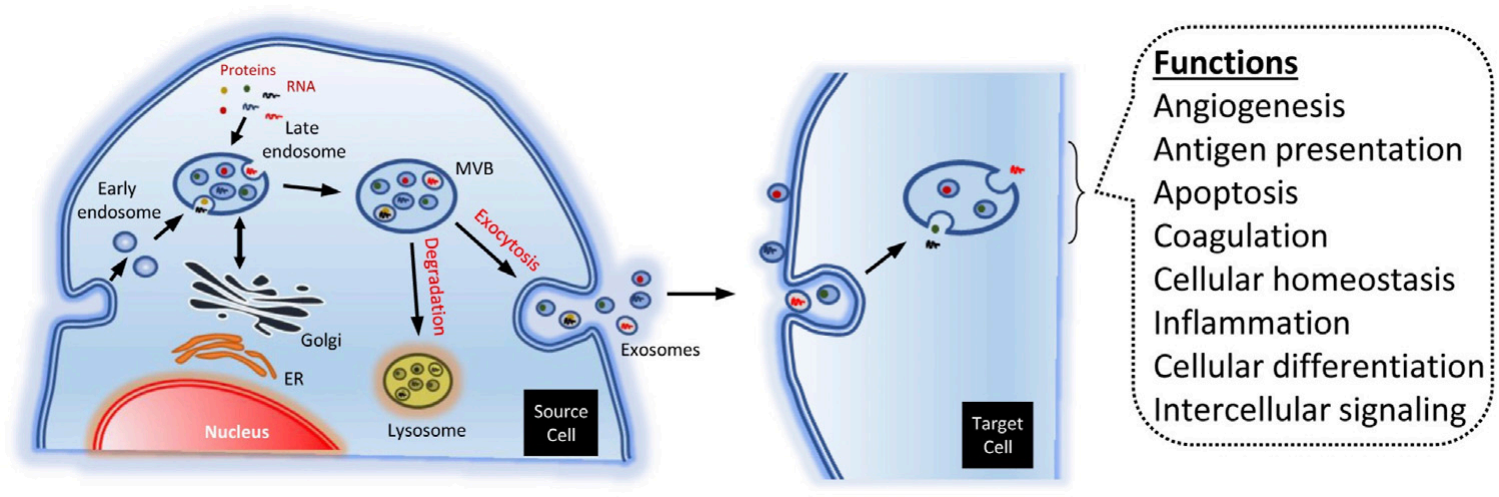
Biogenesis and function of exosomes (Casado-Díaz et al., 2020).

In recent years, there has been extensive research on different types of cells, such as fibroblasts, endothelial progenitor cells, and human umbilical cord mesenchymal stem cells, that are involved in tissue repair by regulating cell function and promoting angiogenesis and wound healing (Zhang et al., 2015a; Li et al., 2016; Geiger et al., 2015; Zhang et al., 2015b). ASC-derived exosomes have also been shown to accelerate wound healing by optimizing fibroblast function (Figure 1) (Ren et al., 2019; Casado-Díaz et al., 2020). Studies have found that ASC-derived microvesicles (ASC-MVs) are easily internalized by human umbilical vein endothelial cells (HUVECs), HaCaTs, and fibroblasts, suggesting that ASC-MVs can serve as a suitable vector for delivering a variety of biomolecules and signals to these targeted cells. ASC-MVs can enhance the migration and proliferation of HUVECs, HaCaTs, and fibroblasts through internalization (Zhang et al., 2018; Bi et al., 2019; Ren et al., 2019). Cell cycle progression can be accelerated in a variety of ways, including by increasing the expression of genes related to cyclin D1, cyclin D2, cyclin A1, and cyclin A2, ultimately promoting wound healing (Bretones et al., 2015).

The migration of HUVECs and angiogenesis play an important role in promoting wound healing. ASC-MVs can significantly upregulate the gene expression of integrin β1 and CXCL16 and regulate migration of HUVECs (Hattermann et al., 2008; Tang et al., 2017). ASC-MVs can also accelerate the wound healing process by promoting angiogenesis (Zhang et al., 2018).

#### ASCs Serve as Effective Immunomodulators in Inflammatory Environments to Promote Wound Healing and Regeneration

Adipose tissue has an immune function because it contains many immune cells and immunomodulatory cells, including ASCs. ASCs regulate mechanisms related to cell differentiation, proliferation, and migration through exosomes by upregulating genes involved in different functions, including skin barrier, immune regulation, cell proliferation, and epidermal regeneration (58). In addition, there are several populations of stromal and immune cells in heterogeneous products obtained after the digestion of adipose tissue, including SVFs. These properties make ASCs effective immune modulators in inflammatory environments (DelaRosa et al., 2012; Gardin et al., 2018; Li and Guo, 2018).

**FIGURE 2:**
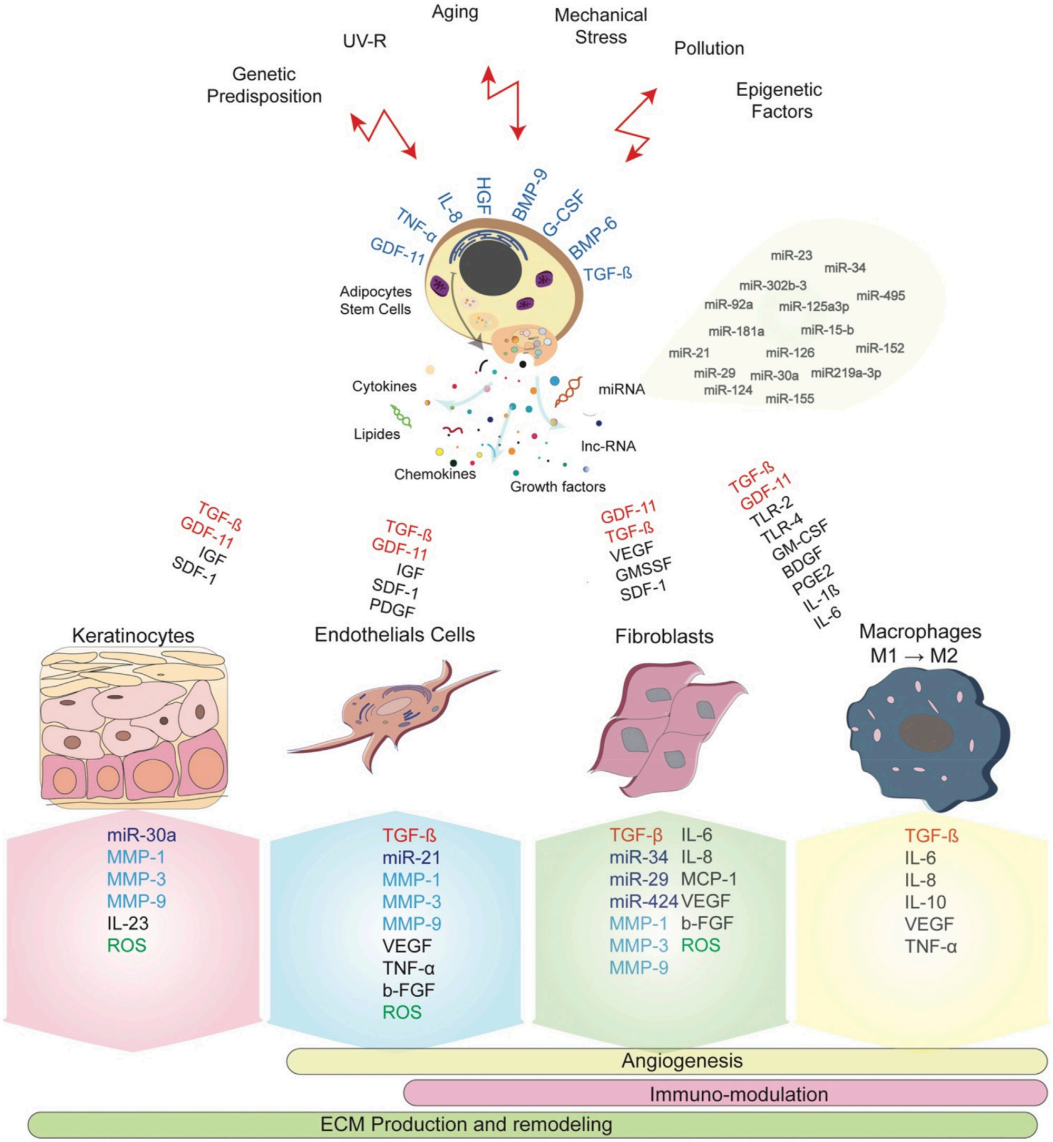
ASCs serve as effective immunomodulators in inflammatory environments to promote wound healing and regeneration (Mazini et al., 2021).

ADSCs directly interact with their microenvironment and specifically the immune cells, including macrophages, NK cell, T cells and B cells, resulting in differential inflammatory and anti-inflammatory effect (Figure 2) (Mazini et al., 2021). The immune regulatory function of ASCs is manifested as regulation of the Th1/Th2 balance and promotion of Tregs to restore immune tolerance. ASCs secrete the anti-inflammatory cytokine interleukin-10 (IL-10), which enhances Treg activity, and Tregs respond by further secreting IL-10 and amplifying IL-10 signaling (Chaudhry et al., 2011). Tregs and IL-10 attenuate Th1 and Th17 activity, thereby reducing the aggregation of additional pro-inflammatory immune cells at pathological sites (Skapenko et al., 2005; Chaudhry et al., 2011). Additionally, the low expression of NK-activated receptor ligands increases human ASC resistance to NK-mediated recognition, which enables them to remain in the host for longer period. Furthermore, the mechanism by which human ASCs develop NK cell tolerance may be mediated by soluble factors (Spaggiari et al., 2008; DelaRosa et al., 2012). The role of these anti-inflammatory and immunomodulatory effects of ASCs in wound healing needs to be further confirmed.

## Discussion

Although ASCs are fundamental to the tissue regeneration process, the clinical transformation of ASC-based therapies remains problematic. Due to the variation in donor age, sex, body mass index, clinical condition, and cell sampling location, ASCs are heterogeneous. Transplanted cells in severe trauma cases have only a limited ability to survive, which can affect their phenotypic features and functions, including proliferation, differentiation potential, immune phenotype, and paracrine activity (Prieto González, 2019). Therefore, future studies on the role of ASCs in regenerative medicine, especially dermatology, are still needed. Nevertheless, ASCs have promising applications in regenerative medicine, including the development of lipogenic potential and the construction of artificial skin by replacing dermal fibroblasts (Trottier et al., 2008; Tartarini and Mele, 2015), which will be the direction of our future research.

## Abbreviations

ASCs, adipose stem cells; ECM, extracellular matrix; IGF, insulin-like growth factor; IL-10, interleukin-10; MVs, microvesicles; PDGF, platelet derived growth factor; SVFs, stromal vascular fragments; TGF-β, transforming growth factor-β.
